# What makes man human: thirty-ninth James Arthur lecture on the evolution of the human brain, 1970

**DOI:** 10.1186/1747-5333-1-13

**Published:** 2006-11-29

**Authors:** Karl H Pribram

**Affiliations:** 1 Professor and head, Neuropsychology laboratories, Stanford University, USA

## Abstract

What makes man human is his brain. This brain is obviously different from those of nonhuman primates. It is larger, shows hemispheric dominance and specialization, and is cytoarchitecturally somewhat more generalized. But are these the essential characteristics that determine the humanness of man? This paper cannot give an answer to this question for the answer is not known. But the problem can be stated more specifically, alternatives spelled out on the basis of available research results, and directions given for further inquiry. My theme will be that the human brain is so constructed that man, and only man, feels the thrust to make meaningful all his experiences and encounters. Development of this theme demands an analysis of the brain mechanisms that make meaning–and an attempt to define biologically the process of meaning. In this pursuit of meaning a fascinating variety of topics comes into focus: the coding and recoding operations of the brain; how it engenders and processes information and redundancy; and, how it makes possible signs and symbols and prepositional utterances. Of these, current research results indicate that only in the making of propositions is man unique–so here perhaps are to be found the keynotes that compose the theme.

## Introduction

**"The hippopotamus may well regard man, with his physical weakness, emotional unpredictability, and mental confusion as a freak.**[1]

**In the middle ages thinkers were trying to discover proofs for the existence of god. Today we seem to look for proof for the existence of man"**[1]

What makes man human is his brain. This brain is obviously different from those of nonhuman primates. It is larger), shows hemispheric dominance and specialization, and is cytoarchitecturally somewhat more generalized [2-6]. But are these the essential characteristics that determine the humanness of man? This paper cannot give an answer to this question for the answer is not known. But the problem can be stated more specifically, alternatives spelled out on the basis of available research results, and directions given for further inquiry.

My theme will be that the human brain is so constructed that man, and only man, feels the thrust to make meaningful all his experiences and encounters. Development of this theme demands an analysis of the brain mechanisms that make meaning–and an attempt to define biologically the process of meaning. In this pursuit of meaning a fascinating variety of topics comes into focus: the coding and recoding operations of the brain; how it engenders and processes information and redundancy; and, how it makes possible signs and symbols and prepositional utterances. Of these, current research results indicate that only in the making of propositions is man unique–so here perhaps are to be found the keynotes that compose the theme.

My concern with meaning originated in an attempt to formulate what ails the current educational process [6,7]. Education entails communication between generations. As such, educational institutions have been set up to transmit information. Our schools have rightly been occupied with problems of information storage and retrieval: what ought to be taught in what period of time and how it is to be demonstrably retrieved. But, it seems this is not enough. From those whom we try to educate we hear rumblings and even shouts of discontent–discontent which arises at least in part from our failure to meet an educational need. What might this be? Is the mere acquisition of information insufficient? May the accumulation of information even be a cause of the problem? Is it not imperative to attempt to impart something additional, something which makes information meaningful?

Information measurement theory provides an interesting starting point for inquiry into this question. In an organism endowed with memory the acquisition of information can, on occasion, actually lead to an increase in uncertainty. Take for instance, a family. The wife is at home, her husband away on a trip, and two children are in college. Her husband informs her that he will call on Thursday, her birthday. Letters from the children give the additional information that they also will call. When the phone rings the wife experiences an amount of uncertainty equivalent to the amount of information she was given initially. She can reduce her uncertainty by obtaining more "information"- asking who's calling. But note that though at the moment of the call the answer to her question provides information, when the extended time period over which the entire episode has transpired is considered, the answer is a repetition of one of the earlier messages. Thus, over time, uncertainty is countered, not by something novel, not by information, but by redundancy, i.e., by repetition. My thesis will be that meaning–the gerund of an old English word for intend, give purpose to–is made possible by repetition. Let me spell out this thesis, first in general, then in brain terms. Repetition comes in many forms. Some forms, some patterns of repetition, are more meaningful than others. Patterns of repetition are called codes. Codes are constructed for a useful purpose. When an organism is uncertain he has two alternative strategies to follow: one, he can reduce uncertainty by seeking real novelty, i.e., information. This, as already noted, will often bring only temporary relief because of man's mnemonic capacity. The other strategy is to reduce uncertainty by coding–by enhancing redundancy, repeating the familiar. This carries the penalty of boredom unless the patterns of repetition are varied. Varying a code turns out to be a remarkably powerful instrument for effectively reducing uncertainty because it permits using information in unexpected ways. From my own research I have concluded that one of the most pervasive–perhaps the most pervasive–of the operations of the brain is, when the need is felt, to actively revise the patterns of redundancy in which information is encoded [8]. There are several levels of these encoding operations, each useful in its own way. Let me first say something about what a code is and then describe the types of codes constructed by the brain.

## What a code is

**"Wonder, or radical amazement, is a way of going beyond what is given in thing and thought. Refusing to take anything for granted, to regard anything as final."**[1]

Not so long ago my laboratory came into the proud possession of a computer. Very quickly we learned the fun of communicating with this mechanical mentor. Our first encounter involved twelve rather mysterious switches which had to be set up (U) or down (D) in a sequence of patterns, each pattern to be deposited in the computer memory before resetting the switches. Twenty such instructions or patterns constituted what is called the "bootstrap" program. Only after this had been entered could we "talk" to the computer–and it to us–via an attached teletype. For example:

DUUUUUDDDDUD

DDDDDDDDUUDD

DUUUDDUDUUUD

DUDDUDDDUUDU

DDUDDDDUUUUU and so on.

Bootstrapping is not necessarily an occasional occurrence. Whenever a fairly serious mistake is made–and mistakes were made often at the beginning–the computer's control operations are disrupted and we must start anew by bootstrapping.

Imagine setting a dozen switches twenty times and repeating the process from the beginning every time an error is committed. Imagine our annoyance when the bootstrap didn't work because perhaps on the nineteenth instruction an error was made in setting the eighth switch. Obviously, this was no way to proceed.

Computer programmers had early faced this problem and solved it simply. Conceptually, the twelve switches are divided into four triads and each combination of up or down within each triad is given an Arabic numeral. Thus,

D D D became 0

D D U became 1

D U D became 2

D U U became 3

U D D became 4

U D U became 5

U U D became 6

U U U became 7

Conceptually, switching the first toggle on the right becomes a 1, the next left becomes a 2, the next after this a 4, and the next an 8. If more than a triad of switches had been necessary, if, for instance, our computer had come with sixteen switches, we should have conceptually divided the array into quads. Thus the bootstrapping program now consisted of a sequence of twenty patterns of four Arabic numerals, such as:

3722

0014

3456

2215

1037 etc.

and we were surprised at how quickly those who bootstrapped repeatedly, actually came to know the program by heart. Certainly fewer errors were made in depositing the necessary configurations–the entire process was speeded and became, in most cases, rapidly routine and habitual. Once the computer is bootstrapped it can be talked to via a teletype in simple alphabetical terms, for example, JMP for jump, CLA for clear the accumulator, TAD for add, etc. But each of these mnemonic devices merely stands for a configuration of switches. In fact, in the computer handbook the arrangement for each mnemonic is given in Arabic notation: e.g. CLA = 7200. This in turn is easily translated into UUUDUDDDDDDD should we be forced to set the switches on the computer by hand because the teletype has gone out of commission.

In the first instance, then, programming is found to be the art of devising codes, codes that when hierarchically organized facilitate learning, remembering and reasoning. The power of the coding process is not to be underestimated. Should you doubt this, try next month to check your bank statement against your record of expenditures and do it all using Roman rather than Arabic numerals. Can you imagine working out our national budget in the Roman system? Next let me turn to an analysis of the classes of codes engendered by the brain. These must account for the existence of subjective states such as perceptions and feelings; for the achievement of acts in the organism's environment; for the construction of signs and symbols by which organisms communicate with each other; and for the composition of propositions, the tools with which man reasons and has fashioned his culture. Research on the brain mechanisms relevant to each of these classes has in recent years yielded some fascinating surprises (Pribram, 1971) [9]. Let me share some of these surprises with you in the search for meaning even if at times the connection between brain, behavior and meaning will appear to be remote. My route is a deliberate one, however, because for me: "Knowing [about meaning has not been] due to coming upon something, naming and explaining it. Knowing has been due to something forcing itself on [me]." [1].

## Brain function in awareness

**"The experience of meaning is an experience of vital involvement. Not an experience of a private reference of meaning, but sharing a dimension open to all human beings."**[1]

During the past decade a series of studies initiated by Kamiya has shown that people can discriminate their brain states [10]. These studies use electrical signals to indicate brain function and recordable behaviors as measures of psychological state. A subject readily acquires the ability to discriminate the occasions when his brain is giving off alpha rhythms from those when his brain's electrical activity is desynchronized. An interesting incidental finding in these studies has been the fact that when Zen and Yoga procedures accomplish their aims, subjects can attain the alpha brain rhythm state at will. Kamiya's training procedures can and are being used as a short cut to Nirvana.

More specific are some recent experiments of Libet that have explored a well-known phenomenon [11]. Since the demonstrations in the late 1800's by Fritsch and Hitzig that electrical stimulation of parts of man's brain results in movement [12], neurosurgeons have explored its entire surface to determine what reactions such stimulations will produce in their patients. For instance, Foerster mapped regions in the postcentral gyrus which give rise to awareness of one or another part of the body [13]. Thus sensations of tingling, of positioning, etc. can be produced in the absence of any observable changes in the body part experienced by the patient. Libet has shown that the awareness produced by stimulation is not immediate: a minimum of a half second and sometimes a period as long as five seconds elapses before the patient experiences anything. It appears that the electrical stimulation must set up some state in the brain tissue and only when that state has been attained does the patient experience.

What do we know about the organization of these brain states apparently so necessary to awareness? They display some curious properties. One would expect that when the brain rhythms which are correlated with the subject's report are disrupted, the behavioral functions would also be interfered with. This is not the case. Focal epileptic discharge in the postcentral gyrus and elsewhere, unless it becomes pervasive and takes over the function of a large part of the brain, does not seriously disrupt awareness [14]. I have densely scattered epileptic lesions in various areas of the nonhuman primate brain in a series of carefully carried out experiments and found that despite the electrical disturbance produced, problem-solving ability remains unimpaired provided the ability had been acquired before electrical seizure discharge began [15-17]. (The acquisition of appropriate performances after the discharges become established is, however, slowed approximately fivefold.)

In short, the brain state necessary to awareness appears to be resistant to being disrupted by local damage provided this damage is not overly extensive. An estimate of the limits on the extent to which disruption can take place without undue influence on the state comes from experiments involving brain tissue removals. Some 85 (or in some experiments even more) of a neural system can be made ineffective without seriously impairing the performances dependent on that system [18-20]. What sort of state is it that can function effectively when only 10 or 15 percent of it remains and all of what remains need not be concentrated in one location?

The answer is that the effective units of the state must be distributed across the tissue involved. Each unit or small cluster of units must be capable of performing in lieu of the whole. Until very recently it was difficult to conceive of such a mechanism.

But just as information processing by computer is an aid in conceptualizing the way in which coding operations are hierarchically constructed, so another engineering domain helps us to understand the problem of the "distributed" state. This domain is called optical information processing [21] because optical systems work this way; or, holography, because each part of a recorded state can stand in for the whole [22].

The essential characteristic of a holographic state is the encoding of the relation among recurrences of neighboring activities. This is known technically as a spatial phase relationship. In optics, ordinary pictures encode only the intensity of illumination at any location; a hologram encodes spatial phase in addition.

Holograms have many properties of interest to the brain scientist. Foremost of these is the fact that information is distributed in the holographic record. Thus one can take a small part of the hologram and reconstruct from it an image in most respects the same as that reconstructed from the whole record. Second, a great deal of information can be stored in one hologram. Several major companies (IBM, RCA) have been able to encode well over a million bits in a square centimeter. Third, an entire image can be reconstructed from a hologram when illumination is reflected from one feature or part of the scene originally recorded. This is the property of associative recall.

**Figure 1:**
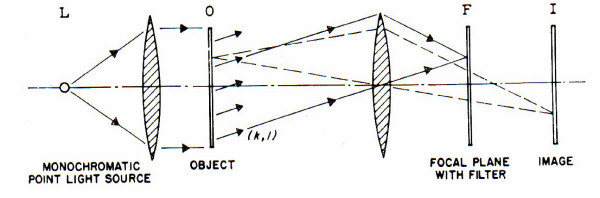
The information to be stored is originally present on a transparent slide in the object plane O. It is illuminated by parallel light from a coherent light source L, like a laser beam. Consequently, in the image plane I one will see an image of the transparent object, faithful within the limitations of the optical system. We now expose a photographic plate, not in I, but in the focal plane F, to the light diffracted by the object. This plate, after exposure, is developed and a positive is made of it, which is put back in F. This filter, which has a transmission in each point proportional to the original light intensity, is called a hologram.

Holograms were first constructed mathematically by Dennis Gabor and crude reproductions were achieved [23,24]. Later they were improved immensely by illuminating the object with a laser beam (fig. 1). Because of the similarity of properties of the optical hologram and the facts about the brain reviewed in the passages above, I have suggested that one important encoding process in the brain follows the mathematical rules of holography [25]. My laboratory is now working on the problem of just how the hologram is realized in neural tissue [7].

The neural hologram is a state in which information is encoded in such a way that images can be constructed. Although images are evanescent, they occur. Although they cannot be directly communicated, they exist. At least three types of images can be discerned subjectively, however, and for each a separate neural system has been identified. Images constructed by the operations of the classical sensory systems refer to events external to the organism [25]; images constructed by the operations of the limbic forebrain monitor the world within [26,27]; and, images constructed by the brain's motor mechanisms structure the achievements an organism aims to accomplish [9, 28]. I want now to take a look at these motor mechanisms, for without them behavior could not occur and we could never make our images meaningful.

## The motor mechanism and acts

**"The deed is the distillation of the self."**[1]

Neuroscientists have engaged in a century-long controversy regarding the functions of the motor cortex of the brain. The view common to all protagonists has been that this tissue serves much as does a keyboard upon which the remainder of the brain–or the mind–constructs the melodies to be executed by muscles as behavior [29]. What has been controversial is the nature of the keyboard. Does it encode, i.e. contain a representation of, individual muscles or even parts [30,31]; or, does the keyboard encode movements, spatial and temporal combinations of muscle contractions, much as do the more complex controls of an organ which encode chords, timbre, etc. [32,33]?

Some years ago I set out to see for myself where I stood in this controversy. I repeated some of the classical experiments and performed others. The results were surprising and I was unable to understand them fully until very recently when additional data from other laboratories became available.

**Figure 2:**
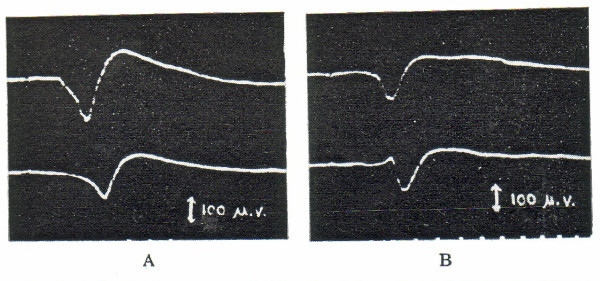
A. Cortical response evoked by stimulation of superficial peroneal nerve. Upper trace in the postcentral "sensory" cortex; lower trace in the precentral "motor" cortex. Time: 10 msec. B. Same as A except that stimulus was applied to posterior tibial nerve. Note that the response in the "motor" cortex is practically identical to that in the "sensory"area.

**Figure 3:**
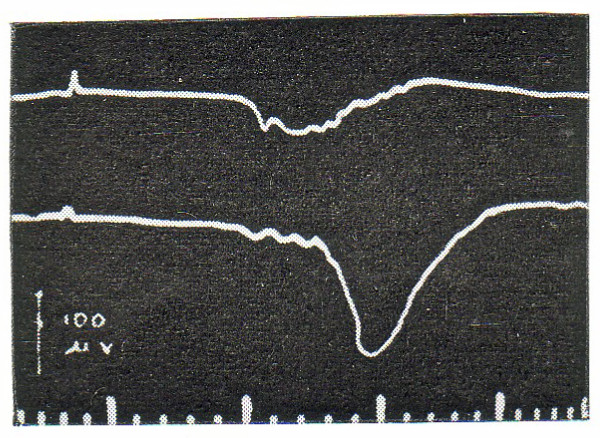
These responses were obtained on sciatic stimulation after complete resection of Cerebellum plus additional resection of cortex of both postcentral gyri. Upper trace, post-Central exposed decorticated white matter; lower trace, precentral cortex. Time: 2 and 10 msec. This indicates that the responses shown in Fig. 2 do not traverse the sensory cortex or the cerebellum on the way to the "motor" cortex.

The first surprise came with the discovery [34] that sensory nerves from both skin and muscle send signals to the motor cortex by pathways no more circuitous than those by which such signals reach sensory cortex (figs. 2 and 3). If the motor cortex were indeed the final common path for cerebral activity, a funnel, what business has it to be informed so directly from the periphery? The problem was compounded by a series of reports of experiments analyzing the organization of peripheral motor control which appeared about this time [35-37]. The results of these experiments showed that one-third of the fibers leaving the spinal cord destined for muscle end in muscle receptors and have, under the experimental conditions, no immediate influence on muscle contraction. What happens when these fibers (called the Y system because they are the smallest in diameter) are stimulated electrically is that a change is produced in the signals going to the spinal cord from the muscle receptors. Until these experiments were reported it had been thought that the signals from the muscle receptors accurately reflected the states of contraction or relaxation of the muscles. Now it became necessary to take into account the fact that messages from the central nervous system could influence the muscle receptors independent of any changes produced in the muscle. The results of both these sets of experiments spelled the end to a simple stimulus-response model of how the nervous system controls behavior [38]. At the periphery the reflex arc became an untenable fiction; at the cortex the keyboard had to give way to some more sophisticated conception.

The second surprise regarding the motor mechanism came with the discovery that I could remove huge amounts of motor cortex with very little impairment of muscle function [28]. Neither individual muscle contractions nor any particular movements were seriously altered by the surgery. Yet something was amiss. Certain tasks were performed with less skill despite the fact that slow motion cinematography showed the movements necessary to perform the task were executed without flaw in other situations. My interpretation of this finding was that behavioral acts, not muscles or movements, were encoded in the motor cortex. An act was defined as an achievement in the environment that could be accomplished by a variety of movements which became equivalent with respect to the achievement. Thus a problem box could be opened by use of a right or left hand; amputees have learned to write with their toes. Encoded in the motor cortex are the determinants of problem solution and of writing–not the particular movements involved in the performance.

What I could not fathom at the time was how the determinants of an act could be encoded. Two experiments have recently helped to clarify my perplexity. One was performed by Bernstein in the Soviet Union [39]. Bernstein photographed people clad in black leotards carrying out preassigned tasks against black backgrounds. Patches of white were attached to the leotards at the locations of major joints. Examples of the tasks are hammering a nail and running over rough terrain. Cinematography showed only the white patches, of course. These described a running wave form which could be analyzed mathematically. From his analysis Bernstein could predict within 2 mm. where the next movement in the action would terminate–where the hammer blow would fall, what level the footsteps would seek. It became obvious that if Bernstein could make such a calculation, the motor cortex could also do it. Interestingly, the equations Bernstein used were the temporal equivalent of those which describe the hologram.

The second experiment gives a clue as to which determinants of acts are encoded [40]. Evarts impaled cells in the motor cortex of monkeys with fine electrodes and recorded the activity of these cells while the monkey pushed a lever. Different weights were attached to the lever so that greater or lesser force had to be exerted by the monkey in order to accomplish the task. Evarts, to his surprise, found that the activity of the cortical neurons from which he was recording varied not as a function of the length or stretch of the muscles used to push the lever but as a function of the force needed to perform the task. Apparently what is encoded in the motor cortex is a representation of the field of forces describing the conditions necessary to achieve an action.

Now the earlier experimental results began to make sense. The motor mechanism resembles a set of thermostats rather than a keyboard [41]. At the periphery the receptors are subject to a dual influence: they are sensitive to muscle tension, which reflects the force exerted on the muscle, and they are sensitive to signals from the central nervous system by way of the Y fibers. This is much like the sensitivity of the thermocouple in a thermostat which is composed of two pieces of metal separated when cool but which make contact with each other by expanding when warmed. In addition to the sensitivity to temperature change the size of the gap between the pieces of metal can be varied by the little wheel at the top of the thermostat–i.e. the device can be set to be more or less sensitive to heat. There is by now a large body of evidence that the Y motor system works by setting the muscle receptor's sensitivity to changes in muscle tension [42]. There is also a great deal of evidence that much of the brain's control over muscle function is performed by making changes in set, in biasing the Y system, and not in making muscles move directly. Note that the setting device of the thermostat is calibrated for temperature, that it has encoded on it the information necessary to control the activity of the furnace to reach the goal set for it and that this goal can be met over a wide range of changes in the temperature of the environment. Note also that the furnace need not display any fixed rhythm of on and off–this rhythm will vary with the environmental exigencies. In the same manner, the brain's motor mechanism can encode the set points, the information necessary to achieve certain acts. The brain need not keep track of the rhythms of contraction and relaxation of individual muscles necessary to achieve an act any more than the thermostat needs to keep track of the turnings on and off of the furnace.

The encoding problem is immensely simplified–only end states need to be specified. As already noted these can be computed by extrapolation from holographic-like equations that summarize the sequence (repetitions) of forces (muscle tension states) exerted.

This is the manner in which the brain achieves acts. But we are not yet arrived at meaning. Acts can be stereotyped, routine. They can be made necessary by environmental change, necessary merely to maintain the organism's equilibrium in the face of such changes. No, there is more to meaning than just action, as there is more to meaning than just imaging. Meaning is derived when acts intend (from the Latin *intendere*, to stretch toward), that is, reach out to, thus impaling otherwise evanescent images and keeping them from slipping away. The brain makes this possible by constructing signs and symbols.

## Signs and symbols: association or differentiation?

**"Knowledge is fostered by curiosity; wisdom is fostered by awe. Awe precedes faith; it is the root of faith."**[1]

Much of my own research on nonhuman primates has been devoted to the problem of how the brain makes possible signs and symbols. For many years I questioned whether, in fact, nonhuman primates could construct signs and symbols but my doubts have now been resolved by work with two chimpanzees, one studied by the Gardners at the University of Nevada [43] and one by Premack at the University of California at Santa Barbara [44]. The Nevada chimpanzee, named Washoe (after the county in which Renois located), has been taught to communicate using a sign language devised for the deaf and dumb. Earlier attempts to set up a rich communicative system between chimpanzee and man had failed. The Gardners felt that this failure was due to the limitations of the chimpanzee vocal apparatus and therefore decided to use a gestural system instead. The system chosen, American Sign Language, has the added feature that it is a relatively iconic rather than a phonetic system, thus much less complex in its structure than is human speech.

Washoe has learned to use approximately 150 signs. She can string two or three signs together but not in any regularly predictable order. Comparison with deaf human children of comparable age shows marked differences in the way in which gestural signs are used–but more of this later. The point here is that sign making is possible for the non-human primate.

The Santa Barbara chimpanzee, Sarah, is being trained by an entirely different method to an entirely different purpose. Premack has taken operant conditioning methods and applied them to determine just how complex a system of tokens can be used to guide Sarah's behavior. Experiments performed in the 1930's had already shown that chimpanzees will work for tokens–in fact a chimpomat had been constructed for use with poker chips. The chimpomat was an outgrowth of the delayed response task, the indirect form of which uses a temporary token to indicate where a piece of food (a reinforcer) is to be found subsequently. The delayed response task had been devised to determine whether animals and children could bridge a temporal gap between a momentary occurrence and a later response contingent on that occurrence. The bridge, which animals and children can construct, has been variously conceptualized in terms of "ideas," "memory traces," "short term memory organization," etc. Premack's chimpanzee has demonstrated that behavior dependent on tokens is not only possible but that hierarchical organizations of tokens can be responded to appropriately.

In all of these experiments the crux of the problem is that the token does not call forth a uniform response. Depending on the situation, that is, the context in which the token appears, the token must be apprehended, carried to another location, inserted into a machine or given to someone, traded for another token or traded in for a reward. Or, as in the original delayed response situation, the token stands for a reward which is to appear in one location at one time, another location at another time.

I shall use the term "symbols" to describe these context dependent types of tokens to differentiate them from "signs" which refer to events independent of the context in which they appear. (This distinction is consonant with that made by Chomsky, "Formal Properties of Grammars," [45] and is used here to indicate that the primordia of the rules that govern human language are rooted in what are here called "significant" and "symbolic" processes.) There is now a large body of evidence to show that the cortex lying between the classical sensory projection areas in the posterior part of the brain is involved in behavior dependent on discriminating signs and that the frontal cortex lying anterior to the motor areas is involved in performances dependent on symbolic processes.

The surprise came when experiments were devised to show how these parts of the brain worked in determining sign and symbol. The ordinary view is that progressively more complex features are extracted or abstracted from information relayed to the projection areas: the simpler extractions occur in the projection areas per se, more complex abstractions demand relays beyond this primary cortex to adjacent stations where associations with information from additional sources (e.g. the primary projection areas) are made available [46]. Unfortunately for this view there is a good deal of experimental evidence against it.

**Figure 4:**
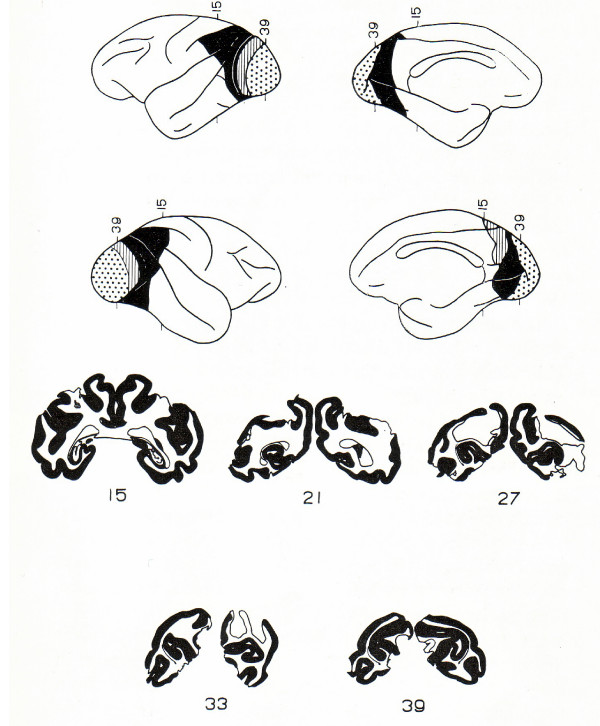
Diagrammatic reconstruction of the brain after an essentially complete lesion of the Peristriate cortex. Representative cross sections are shown by number indicating placement on brain diagram. The monkey from whom this brain was taken retained a visual discrimination habit perfectly.

Most direct is the fact that if progressive cortico-cortical relays are involved in the ability to utilize signs and symbols, then removals of these relays should impair the ability. This is not the case. The posterior and frontal cortices specifically concerned in sign discrimination and in delayed response lie some distance from the primary sensory and motor areas. Complete removal of the tissue that separates the primary areas from those involved in discrimination and delayed response does not permanently impair the performance of these tasks: Fig. 4[20,47,48]. Ergo, cortico-cortical "abstractive" relays cannot be the mechanism at issue.

Two possibilities remain to explain the involvement of those cortical areas remote from the primary projection zones in discrimination and delayed response behavior. Information may reach these areas by routes independent of those that serve the primary projection cortex. This possibility is being actively explored in several laboratories. In the rhesus monkey, however, there is already evidence that these independent routes do not play the desired role: destruction of the pathways does not lead to a deficit in the performance of discriminations or delayed response [49,50].

**Figure 5:**
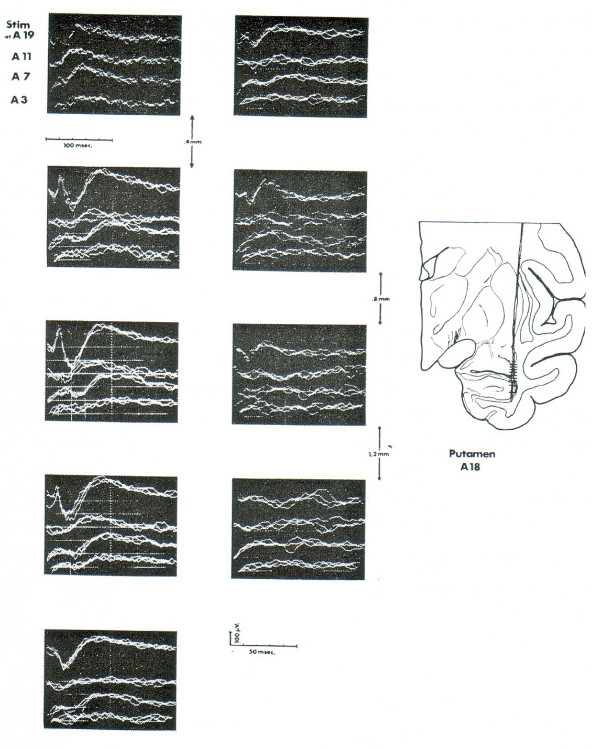
Responses evoked by stimulation of the part of the temporal lobe involved in vision. Note tracts passing through the putamen, one of the major motor structures in the brain. Horizontal marks indicate the location of the tip of the recording electrode from which the Response was photographed.

The third possibility is one that I have been seriously exploring for the past decade and a half [51]. This alternative holds that sign and symbol are constructed by a mechanism that originates in the cortex and operates on the classical projection systems in some subcortical location. Thus the effects of the functioning of the cortex involved in signing and symbolizing are conceived to be transmitted downstream to a locus where they can preprocess signals projected to the primary sensory and motor cortex. A good deal of evidence has accrued to this third alternative. Perhaps most important is the fact that a large portion of the pathway relays within the basal ganglia, motor structures of the motor mechanism of the brain:Fig 5[52]. Sign and symbol manipulation thus involves the same brain structures that are used by the organism in the construction of acts. The suggestion that derives from these anatomical facts is that signifying and symbolizing are acts, albeit acts of a special sort.

There is, of course, a difference in the neuroanatomy involved in signifying and that involved in symbolizing. This difference, as well as the behavioral analysis of the tasks involved, tells a good deal of what these behavioral processes are all about. The pathways for signifying influence the primary sensory systems. Connections have been traced by electrophysiological techniques as far peripheral as the retina [53,54] and the cochlear nucleus [55], for instance. The connections important to the symbolic process have not as yet been determined as fully, but a good deal of the evidence points to involvement with the limbic systems structures on the innermost boundary of the forebrain [56].

This connection between limbic and frontal lobe function demands a word or two. Removal of tissue in these systems does not impair sign discrimination but does impair performance on such tasks as delayed alternation [57-59], discrimination reversal [60], shuttle-box-avoidance [61] and approach-avoidance, commonly called "passive" avoidance [62]. In all of these tasks some conflict in response tendencies, conflict among sets, is at issue. The appropriate response is context (i.e. state) dependent and the context is varied as part of the problem presented to the organism. Thus a set of contexts must become internalized (i.e. become brain states) before the appropriate response can be made. Building sets of contexts depends on a memory mechanism that embodies self-referral, rehearsal or, technically speaking, the operation of sets of recursive functions. (The formal properties of memory systems of this type have been described fully by Quillian [63]. The closed loop connectivity of the limbic systems has always been its anatomical hallmark and makes an ideal candidate as a mechanism for context dependency [64,65].

As an aside, it is worth noting that much social-emotional behavior is to a very great extent context dependent. This suggests that the importance of the limbic formations in emotional behavior stems not only from anatomical connectivity with hypothalamic and mesencephalic structures but also from its closed loop, self-referring circuitry. It remains to be shown (although some preliminary evidence is at hand [66,67] that the anterior frontal cortex functions in a corticofugal relation to limbic system signals much as the posterior cortex functions to preprocess sensory signals.

Thus signs and symbols are made by the brain's motor mechanism operating on two classes of images–in the case of signs those that encode sensory signals and in the case of symbols those that monitor various states of the central nervous system. Signs are codes invariant in their reference to events imaged–their meaning is context free. The meaning of symbols, on the other hand, is context dependent and varies with the momentary state induced in the brain by the stimulation. Both signs and symbols convey meaning, make possible a temporal extension of otherwise momentary occurrences.

Man shares the meaning conveyed by sign and symbol with nonhuman animals. This form of meaning, though perhaps more highly developed in man than in other animals, is not what makes him peculiarly human. Our search for man's unique thrust to make all his experiences and encounters meaningful needs to proceed to yet another level of complexity of encoding: only man makes propositions and reasons with them.

## Propositions and reasoning: using signs symbolically and symbols significantly

**"Man may, indeed, be characterized as a subject in quest of a predicate, as a being in quest of a meaning of life, of all life, not only of particular actions or single episodes which happen now and then." **[1]

A proposition is a sentence. It is made up of nouns and a predicate. Nouns are derived from signs; nouns can be conceived as signs used in sentences. Verbs are not so easy to characterize. Most verbs are also derived from signs; verbs indicate actions instead of things. Adjectives and adverbs also display this property of signification. Thus cow, green, grass, run, chew, stand, trough, drink, water, are all signs depicting events and occurrences. Only when used in sentences do these signs become nouns, verbs, adjectives and adverbs. What then makes a sentence?

Sentences are codes constructed by the mechanism of predication. My hypothesis is that predication is a symbolic process, i.e. it places linguistic signs into a context dependent frame. Predication depends on the verb "is" in its various grammatical constructions and according to my hypothesis all basic sentences are explicitly or implicitly of the form "X is Y."

As a corollary, predication is conceived to be a statement of belief. (See Ayer, 1946, pp. 8–15 and 91–93, for similar views [68]). The maker of a proposition is communicating his belief with regard to a relationship among signs. Thus negation, qualification and the like are part of predication. The sentence "the boy runs" is therefore a shorthand statement of the sentence "the boy is running" and indicates certainty on the part of the speaker. "I believe the boy is running"; "I think the boy is running"; "the boy may or may not run" are all qualifiers on the certainty with which the proposition is held. It is this process of making statements of certainty of belief that is unique to man and provides the thrust toward making experiences and encounters meaningful. Propositions power meaning by introducing flexibility into the relationship among signs. A new level of coding emerges, the best formal example of which is the alphabet. Each letter is a linguistic sign, a context-free indicator that can be used as such–for instance, in organizing a dictionary. The symbolic use of the alphabet, on the other hand, provides an infinite richness of meaning through combinations of the self-same letters where context dependent relationships now become paramount. Thus "tap" and "pat" have different meanings.

Man not only uses linguistic signs symbolically, he uses linguistic symbols significantly. This he does when he reasons. He takes a context dependent linguistic symbol and for the duration of a particular purpose assigns to it a context-free meaning. This is accomplished by making explicit a set of rules governing the relationship among linguistic symbols "for the duration." The set of rules is, of course, a set of propositions. Algebra is probably the most familiar formal example of reasoning.

The point at issue is that though animals make signs and symbols, only man appears to use linguistic signs symbolically in making propositions and linguistic symbols significantly in reasoning. What then is different about man's brain that makes possible a reciprocal interaction between sign and symbol?

The common answer to this question is that man's brain is characterized by its massive cortico-cortical connectivity [69]. This connectivity is conceived to be quantitatively, not qualitatively, different from that of non-human brains. But as we have already seen, the postulated transcortical relay mechanism of sign and symbol construction does not come off well when examined in the light of experimental evidence obtained with nonhuman primates. Instead, signs and symbols are found to be made by virtue of a mechanism that involves cortico-*sub*cortical connections that relay in structures hitherto conceived to be motor in function. Thus if man's special capability is due to his brain's cortico-cortical connectivity, this difference is qualitative not just quantitative.

The issue is an important one. If, in fact, the cortico-cortical connectivity of man's brain proves to be the source of his power of prepositional language and reasoning, we have an answer to the question of what makes man human. A great deal is being made today of this cortico-cortical connectivity in terms of the "disconnection" syndromes that result in a variety of aphasias and agnosias. But data from the clinic are not always easy to evaluate and misinterpretation due to unqualified preconceptions can readily occur. I have some misgivings about the validity of the common view that cortico-cortical connections are responsible for man's human capabilities. I cannot now fully spell out these misgivings because they are intuitive and constitute the questions directing my research plans for the immediate future. But a few points can be made. Obviously the roots of the misgivings lie in my experience with nonhuman brains. Initially the cortico-cortical hypothesis seemed self-evident. Only when experimental result after experimental result disconfirmed the hypothesis was I driven to search elsewhere to make sense of the data. However, this is not all. The cortico-cortical connection hypothesis implies that information is transmitted by the connections. The largest bundle of connecting fibers, and one that has grown considerably in size when man is compared to monkey, is the corpus callosum which connects the two hemispheres. Yet this increase in the connectivity between hemispheres in man has led to hemispheric specialization, each hemisphere serving widely different functions. The connections seem to make it possible for the hemispheres to go their separate ways to a large extent rather than to duplicate each other as they do in non-human mammals [70,71].

Objections to this view of the functions of the corpus callosum immediately come to mind as a result of Sperry's fascinating split-brain patients [72]. Sperry demonstrates that each hemisphere can be shown to control awareness independent of the other hemisphere once the callosum is cut. He infers from this that separate consciousnesses, separate minds, exist in one head in these patients. The assumption underlying this inference is that ordinarily consciousness is of a piece and that we are always single-minded. I challenge this assumption. Single-mindedness is an achievement that often demands considerable effort whether one is studying, listening during a conversation, or driving an automobile. Sperry's patients are not unique in being of two minds on occasion.

Other evidence that gives rise to my misgivings with the connectionist hypothesis comes from unilateral brain ablations that produce symptoms which are alleviated by further brain ablation. Thus unilateral ablations of the frontal eye-fields in monkey and man result in a temporary disregard of stimuli in the contralateral visual field [73,74]. Such disregard does not occur if the lesion is bilateralized. Also, unilateral occipital lobectomy in the cat results in a homonymous hemianopia which is relieved when the ipsilateral optic colliculus is removed [75].

These are but straws in the wind but they prevent me from obtaining too easy and early a closure on the problem of what makes man human. In order that the issue can be faced squarely, however, I must offer an alternative to the cortico-cortical connection hypothesis. My alternative is that man makes meaning through signs, symbols, propositions and reasoning by way of corticofugal-subcortical connections that importantly involve the motor mechanisms of the brain. I propose that man's thrust toward meaning derives from the fact that his brain's motor mechanisms are better developed than those of animals. These motor mechanisms are not to be conceived, as we have seen, merely as movers of muscles. The brain's motor mechanisms are devices that set the sensitivity of receptors and afferent channels, not just of muscle receptors but those of all receptors (including eye and ear) as well. Changes in setpoint regulate awareness and behavior. The changes and their results can relatively simply be encoded in brain tissue and thus serve as guides subsequently.

## Conclusion

**"Thinking is living and no thought is bred in an isolated cell in the brain." **[1]

The implications for education of this propensity of the brain for encoding and receding its sensitivities are obvious. In order to make information meaningful we must allow pupils to encode in terms of their own sensitivities which are not necessarily ours. They must be given the opportunity to repeat the information given in such a way that it becomes encoded in a context which makes meaning for them. They must be encouraged to remake what we give them in their own image.

This is not as difficult as it sounds. As already noted, even young children who are deaf use signs differently from the way Washoe the chimpanzee uses signs. Human children spontaneously make propositions, their language is productive (Jakobsen, 1966) [76]. All neural tissue is spontaneously active, nerve cells beat out electrical signals on their own throughout life, much as does the tissue of the heart. In man this spontaneity becomes organized early on so that he produces propositions, makes sentences. And then he begins to play with these sentences, receding them into different forms and reasoning with them. Each new batch of teenagers attests to the human proclivity for productively receding what is given. Why not utilize this marvelous capacity to advantage in our educational effort?

To summarize briefly: man's brain is different in that it makes imperative the productive use of linguistic signs symbolically and linguistic symbols significantly. The flexibility derived from this difference is immense. Given the power of this flexibility man codes and recedes for fun and profit. Every artistic endeavor, every working accomplishment depends for its effectiveness not only on the information conveyed by the theme but on the variations on that theme. Human encounter is sustained not just by an exchange of information but by an infinite variety in familiar communication. Animals use signs and symbols only in special circumstances; man productively propositions all his encounters and he reasons about all his experiences. Thus man and only man shows this thrust to make meaningful his experiences and encounters: he intends, he holds on to his images.

But this is not all. By means of the motor mechanisms of his brain man hopefully and continuously sets and resets his sensitivities so that his images can become actualized in his environment both by virtue of his own behavior and that of socially contiguous others. Man's culture expresses these hopes, this active thrust toward meaning. For to be human is to be incapable of stagnation; to be human is to productively reset, reorganize, recode, and thus to give additional meaning to what is. In short, "to be human is to be a problem." [1].

## Note

Many important aspects of the problem of the brain's coding processes are dealt with here altogether too briefly. But the present paper will serve as a prolegomenon to a more comprehensive study which will appear under the title *Languages of the Brain: Experimental Paradoxes and Principles in Neuropsychology*, to be published by Prentice-Hall in 1971 [9].

## Other James Arthur lectures on the evolution of the human brain

Frederick Tilney, The Brain in Relation to Behavior; March 15, 1932

C. Judson Herriek, Brains as Instruments of Biological Values; April 6, 1933

D. M. S. Watson, The Story of Fossil Brains from Fish to Man; April 24, 1934

C. U. Ariens Kappers, Structural Principles in the Nervous System; The Development of the Forebrain in Animals and Prehistoric Human Races; April 25, 1935

Samuel T. Orton, The Language Area of the Human Brain and Some of its Disorders; May 15, 1936

R. W. Gerard, Dynamic Neural Patterns; April 15, 1937

Franz Weidenreich, The Phylogenetic Development of the Hominid Brain and its Connection with the Transformation of the Skull; May 5, 1938

G. Kingsley Noble, The Neural Basis of Social Behavior of Vertebrates; May 11, 1939

John F. Fulton, A Functional Approach to the Evolution of the Primate Brain; May 2, 1940

Frank A. Beach, Central Nervous Mechanisms Involved in the Reproductive Behavior of Vertebrates; May 8, 1941

George Pinkley, A History of the Human Brain; May 14, 1942

James W. Papez, Ancient Landmarks of the Human Brain and Their Origin; May 27, 1943

James Howard McGregor, The Brain of Primates; May 11, 1944

K. S. Lashley, Neural Correlates of Intellect; April.30, 1945

Warren S. McCulloch, Finality and Form in Nervous Activity; May 2, 1946

S. R. Detwiler, Structure-Function Correlations in the Developing Nervous System as Studied by Experimental Methods; May 8, 1947

Tilly Edinger, The Evolution of the Brain; May 20, 1948

Donald 0. Hebb, Evolution of Thought and Emotion; April 20, 1949

Ward Campbell Halstead, Brain and Intelligence; April 26, 1950

Harry F. Harlow, The Brain and Learned Behavior; May 10, 1951

Clinton N. Woolsey, Sensory and Motor Systems of the Cerebral Cortex; May 7, 1952

Alfred S. Romer, Brain Evolution in the Light of Vertebrate History; May 21, 1953
